# A Journey Towards Meaning: An Existential Psychobiography of Maya Angelou

**DOI:** 10.5964/ejop.5491

**Published:** 2021-08-31

**Authors:** Nadene Harisunker, Carol du Plessis

**Affiliations:** 1 Department of Psychosocial Health, North West University, VTC, Gauteng, South Africa; 2 School of Psychology and Counselling, University of Southern Queensland, Ipswich, Australia; Nelson Mandela University, Port Elizabeth, South Africa

**Keywords:** psychobiography, Maya Angelou, Victor Frankl, existential psychology, meaning making, fundamental triad

## Abstract

This psychobiography focuses on meaning making in the early life and young adulthood of acclaimed African American author Maya Angelou (1928-2014) through the lens of Frankl’s existential psychology with a specific focus on the tri-dimensional nature of human beings and the fundamental triad. The primary data source was Angelou’s own published autobiographies, which contain an in-depth narrative of her early life and young adulthood. Data was extracted, organised and analysed according to established qualitative research methods as well as through the identification of psychological saliences. The search for meaning within Angelou’s own narrative of her life was clearly apparent in the thematic analysis. Angelou’s narrative of her journey through the physical (childhood and adolescence), psychological (travelling and searching years) and spiritual (sensemaking years) dimensions was core to her meaning making. The three tiers of the fundamental triad (awareness of meaning, will to meaning, freedom of will) were present in various aspects of Angelou’s existential journey, manifesting as a focus on choice, responsibility, purpose, and acceptance. This study provides a more in-depth understanding of meaning making processes in the lives of extraordinary individuals, as well as contributing to the development of the research method of psychobiography, with a specific focus on meaning making.

This psychobiographical study explores meaning making processes in the early life and young adulthood of Maya Angelou, an acclaimed African American female poet, author, playwright, and teacher. Angelou chronicled this period of her life through her extensive autobiographies containing personal commentary and reflexive engagement with her socio-historical milieu (Angelou, 2004). The books, beginning with *I Know Why the Caged Bird Sings* (Angelou, 2004), span the first 40 years of her life and are used in this analysis to chart her engagement with, and relationship to, meaning. As such, this analysis focuses on the first 40 years of her life, in keeping with the focus in her autobiographies.

## Maya Angelou: A Brief Biography

Maya Angelou was born Marguerite Ann Johnson on the 4th of April 1928. Her parents separated soon after her birth (Agins, 2013; Angelou, 2004; Lupton, 1998) and Angelou spent her early childhood years living with her paternal grandmother (Momma) in Arkansas (Agins, 2013; Angelou, 2013; Lupton, 1998). Momma provided a strict religious upbringing that was devoid of any overt expressions of love – both physical and verbal (Angelou, 2004, 2013).

In 1934, when Angelou was 6 years old, she moved to live with her mother. Angelou reported experiencing low self-esteem and poor body image and she frequently compared herself unfavourably to her beautiful mother, brother, and father (Angelou, 2004; Lupton, 1998). In 1935 Angelou was sexually abused and subsequently raped by Mr F., her mother’s boyfriend. When Angelou told her uncles about her abuse, they responded by beating Mr F. to death. In her autobiography Angelou notes that she came to believe her voice was deadly and poisonous, and she became selectively mute for several years.

Angelou moved between living with her mother, grandmother, and father throughout her teenage years (Agins, 2013; Lupton, 1998). Although she excelled academically this was also a time of tumult. For example, in 1943 she spent a month living in a junkyard with fellow teenagers after a physical altercation with her father’s girlfriend. Angelou also became pregnant as a teenager and her son, Guy, was born in 1945.

Following her graduation from high school, Angelou worked at various jobs. She was insecure and acted out in ways she believed would help her fit in (Angelou, 2004). Two specific incidents are highlighted in her autobiographies: once when she prostituted herself for her boyfriend in order to ensure his continued investment in her and once when she argued with a white staff member at a shop due to her need to prove herself superior (Angelou, 2004). During this time Angelou also experienced frequent anxiety and guilt concerning her role as a mother as she felt tension between her desire to be a perfect mother and her desire to explore her career (Angelou, 2004).

Relationships also played an important role in Angelou’s development during this time. In her autobiographies, she notes that she often became submissive and changed who she was in order to be accepted by her partner. In 1953 she married Tosh Angelos, a white man with whom she believed she could have a perfect life. However, she failed to find meaning and purpose in the relationship, and when the marriage ended she rediscovered a love for dance that characterised the next ten years of her life. Angelou worked as a professional dancer, addressing her insecurities and chafing against the control of a second, fairly short, unofficial marriage to Vus, a South African freedom fighter. Following the end of this relationship Angelou moved to Ghana where she grappled with her ‘Africanness’ and eventually accepted her African American identity (Angelou, 2004, 2013).

Once she returned to America in 1965, Angelou continued to move frequently and her dance career gradually gave way to a career in political activism and writing. She became a successful playwright and author with a career spanning the next five decades. One of her major contributions as an author was a six volume autobiography, published between 1969 and 2002, charting the first 40 years of her life prior to her career as a writer. These volumes, as well as a seventh volume that focuses on her relationship with her mother and grandmother, are the primary data set for this study.

Angelou died on May 28th 2014 at 86 years of age. Her legacy includes her incredible corpus of writing as well as significant contributions to the civil rights movement and to recognition for female black authors. She has been described as a warm, kind, generous, powerful, and phenomenal individual (Cadet, 2014; Snow, 2014) and her main message was that “[p]eople must work to overcome their hardships with dignity and view the world with hope and love” (Agins, 2013, p. 101).

The Multilayered Chronological Chart (MCC; see Hiller, 2011) in Table 1 below captures core moments in Angelou’s life in accordance with various themes evidenced in her narrative – specifically relationships and career. It also illustrates the time period covered by each of the first six volumes^1^1Note that there is a 7th volume, *Mom & Me & Mom,* published in 2013, but this does not relate to a specific time period but instead focuses on her relationship with her mother and grandmother. of her autobiography.

**Table 1:** Multilayered Chronological Chart: Maya Angelou

Childhood (1928–1940)	Teenage years (1941-1945)	Searching for escape/pleasure (1945-1955)	Travelling and self discovery (1955–1965)	The sense making years (1965-2014)
Biographical events				
1928: Born 1931: Moved to Arkansas 1933: First contact with parents 1935: Moved to live with mother 1935: Molested and raped by Mr F. 1935: Mr F. was beaten to death by Maya’s uncles 1935–1940: Selectively mute 1935: Returned to Arkansas	1941: Returned to live with mother 1943: Lived briefly with father until she was stabbed by step-mother and lived on the streets for 1 month	1945: Returned to Arkansas 1946: Returned to live with mother 1948: Guy kidnapped 1953: Death of grandmother 1953: Began to use the name Maya	1955–1957: Moved several times within USA 1962: Moved to Cairo 1962: Moved to Ghana 1962: Guy was involved in a serious car accident 1965: Returned to USA 1965: Guy was involved in another car accident	1966: Looked after Guy 1967: Returned to New York 1982: Moved to North Carolina 1990: Mother diagnosed with Cancer 1992: Mother passed away
Relationships				
	1945: Birth of son (Guy)	1948: Dated an older man and agreed to work as a prostitute for him 1953: Married Tosh (marriage lasted 2 years)	1961: Verbally married Vus (South African freedom fighter) 1962: Relationship with Vus ended	1973: Married Paul de Feu 1981: Divorced Paul de Feu
Career				
1940: Graduated top of her class	1943: Became first Black Female Street Conductor 1945: Graduated Highschool	1948: Worked briefly as a prostitute, along with other insecure jobs 1953: Began a career as a dancer	1954-1955: Two year dance tour of Europe 1956: Worked as a singer, became interested in writing 1957: Joined Harlem Writers Guild	1967: Decided to focus on a career as a writer **Received many accolades, including:** 1971: Nominated for Pulitzer Prize 1973: Acted in a Broadway Play 1976: Nominated for an Emmy 1981: Appointed Reynolds Professor in American Studies 1993: Recited poem at US Presidential Inauguration
Autobiography				
1928-1945 *I know why the caged bird sings* (published 1969)	1946–1947 *Gather together in my name* (published 1974)	1945–1955 *Singing’ and Swinging’ and Getting’ Merry like Christmas* (published 1976)	1952–1957 *The Heart of a Woman* (published 1981) 1962–1965 *All God’s Children need travelling shoes* (published 1986)	1965–1968 A *song flung up to Heaven* (published 2002)

## Theoretical Framework: Frankl’s Existential Psychology

This study explores meaning making using Frankl’s tri-dimensional view of humans as well as his fundamental triad, with a focus on a striving to meaning and meaning through struggle (Bruner, 2012). Frankl’s theory, developed in the 1920s and refined through his experiences in the Nazi concentration camps, is existential in nature and emphasises individual choice and meaning making. Frankl’s theory highlights major concepts that are fundamental to meaning making such as an awareness of the spiritual dimension, the responsibleness to meaning, and the purposeful nature of meaning making (Wong, 2014). The core aspects of Frankl’s theory used in this psychobiography are discussed below.

### Tri-Dimensional View of Humans

Within Frankl’s theory, humans are viewed as tri-dimensional, consisting of a biological/physical body, the psychological/inherited self and finally, a noetic (spiritual) dimension. For Frankl, the ‘noos’ or ‘mind’ constitutes the very being of humans (Wong, 2014). While Frankl accepts that there are drives (biological/physical and psychological) within humans and that societal circumstances impact individuals, within his theory the spiritual ‘noetic’ self is in control over and above these (Morgan, 2012). The spiritual dimension is a defiant power within humans that provides for transcendence from both instinctual/internally and externally determined sources and allows for meaning making (Ras, 2000). Within this spiritual dimension, meaning making is unique to each individual, and each individual experiences unique obstacles to meaning making, which usually occur when the spiritual dimension is ignored and conflicts arise. These must be engaged with to ensure meaning making in a human life.

### Fundamental Triad

The fundamental triad forms the basis of meaning and consists of three facets: an awareness of the existence of meaning, a will to meaning, and a freedom to will. These are all inherent to the noetic dimension of human beings and are all interlinked (Mun, 2005). An awareness of the existence of meaning is the first aspect of the fundamental triad. The spiritual core within all individuals enables the process of awareness, allowing for the discovery of meaning. Once there is awareness of a spiritual sphere, there is a striving for meaning and therefore a realisation that meaning exists in the world. Meaning can never be created but only discovered through indirect means. Therefore, all that people can do is pursue what fulfils them and gives them a greater purpose. In addition, the discovery of meaning comes as a result of extending beyond the individual self through the acknowledgement of social reality and others within this reality. Meaning exists in every moment of an individual’s life; it is the individual’s responsibility to discover these meaning moments (Frankl, 1969).

The second aspect of the fundamental triad is the will to meaning, and it is this feature that makes every individual unique as they strive to discover their own meaning in life. A pivotal tenet of Frankl’s theory is the motivating and striving force of the human spirit (Frankl, 1967, 1969). The will is a driving force; defined by its future orientation, its inclination away from self-absorption and the movement towards experiencing meaning outside of the self (Meyer, 1997). The will to meaning is defined by self-awareness and a way of being that is inclined towards growth. Conflicts arise but they are necessary and contribute to the discovery of meaning. An individual can be understood through the situations and experiences that they find meaningful and the way in which they direct their energy to discover meaning (Frankl, 1967).

The third component of the fundamental triad is the freedom to will. Every individual has the freedom to make choices in their lives that lead to the discovery of meaning. Freedom to will lies in the responsibleness of a person: Responsibleness is within the internal locus of control of the individual while responsibility entails an obligation that is imposed from outside the individual (Fabry, 1987). An individual is not driven to meaning but is free to make choices. Individuals are often unaware that allowing their circumstances to control them is making a choice to relinquish their freedom (Fabry, 1987; Frankl, 1965, 1985, 2000).

## Aim of the Present Study

The primary aim of this study was to explore meaning making in the early life and young adulthood (until approximately age 40) of Maya Angelou, through the lens of Frankl’s existential framework. This aim was addressed by meeting the following specific objectives:

1. Developing a psychological portrait of Maya Angelou using psychobiography methods and an MCC. This portrait focused on her development until the age of 40.
2. Explore meaning making in the early life and young adulthood of Maya Angelou through the lens of Frankl’s tri-dimensional view of human nature as well as the fundamental triad.

## Method

Psychobiography aims to develop an understanding of an individual life through the application of a theoretical framework (Ponterotto, 2013, 2014; Runyan 2005; Schultz, 2005). Psychobiography involves both case study and narrative research (Schultz, 2005). The theoretical framework provides a lens through which to view a biographical account from a psychological perspective in order to arrive at a greater understanding of an individual life (Elms, 1994). In order to ensure credibility and trustworthiness, all psychobiographies follow rigorous methods of data collection and data analysis, and follow certain ethical guidelines. These processes are discussed in detail below.

### Data Collection

Angelou produced extensive autobiographies detailing her life story from age three to age 40. These autobiographies, published in seven volumes, constituted the primary source of data for this analysis (Angelou, 2004, 2013). Information regarding the remainder of her life was accessed through secondary sources, including biographies and critical literary works. However the primary focus of the analysis was on the earlier years of her life, as this is the period presented in her autobiographies. Ethical approval was not sought for this study as the data used is in the public domain and the subject of the psychobiography is deceased. However, ethical guidelines specific to psychobiography were adhered to including ensuring that no confidential information was disclosed in the published psychobiography and ensuring that the psychobiographical subject was treated with respect and dignity (Ponterotto & Reyonlds, 2017).

### Data Extraction

The data extraction process for this psychobiography followed the guidelines provided by Miles, Huberman, and Saldaña (2014). In the first step, data condensation was used to reduce the volume of data. Two methods were selected to condense data into meaningful units: Alexander’s indicators of psychological saliency and Schultz’s prototypical scenes (Schultz, 2005). Both techniques comprised a framework used to elicit the psychologically salient aspects present within the data to ensure that valid conclusions were drawn (Perry, 2012; Schultz, 2005). Data identified as psychologically salient was extracted from the primary data sources and collated into a single document. The data was then organised so that patterns and themes could be identified. Data was first organised in a chronological fashion and this chronology forms the basis of the MCC (Hiller, 2011) presented in the introduction to this paper.

### Data Analysis

A deductive thematic analysis was conducted, where the two triads from Frankl’s theory (the view of a person and the fundamental triad) were used to identify themes in the data. This resulted in the identification of themes that were directly related to the concepts within Frankl’s theory. In order to ensure that themes were not artificially imposed on the data, Runyan’s (1981) criteria for good psychobiographical interpretation were applied to the analysis. This deductive thematic analysis process was cyclical in nature as there was a continuous vacillation between data and conclusions to ensure trustworthiness and quality of the research (Miles et al., 2014).

## Findings and Discussion

The analysis process resulted in the identification of all aspects of Frankl’s triads within Angelou’s chronological narrative in the first 40 years of her life. In the sections below the two triads are discussed separately.

### Tri-Dimensional View of Humans

**Figure 1:**
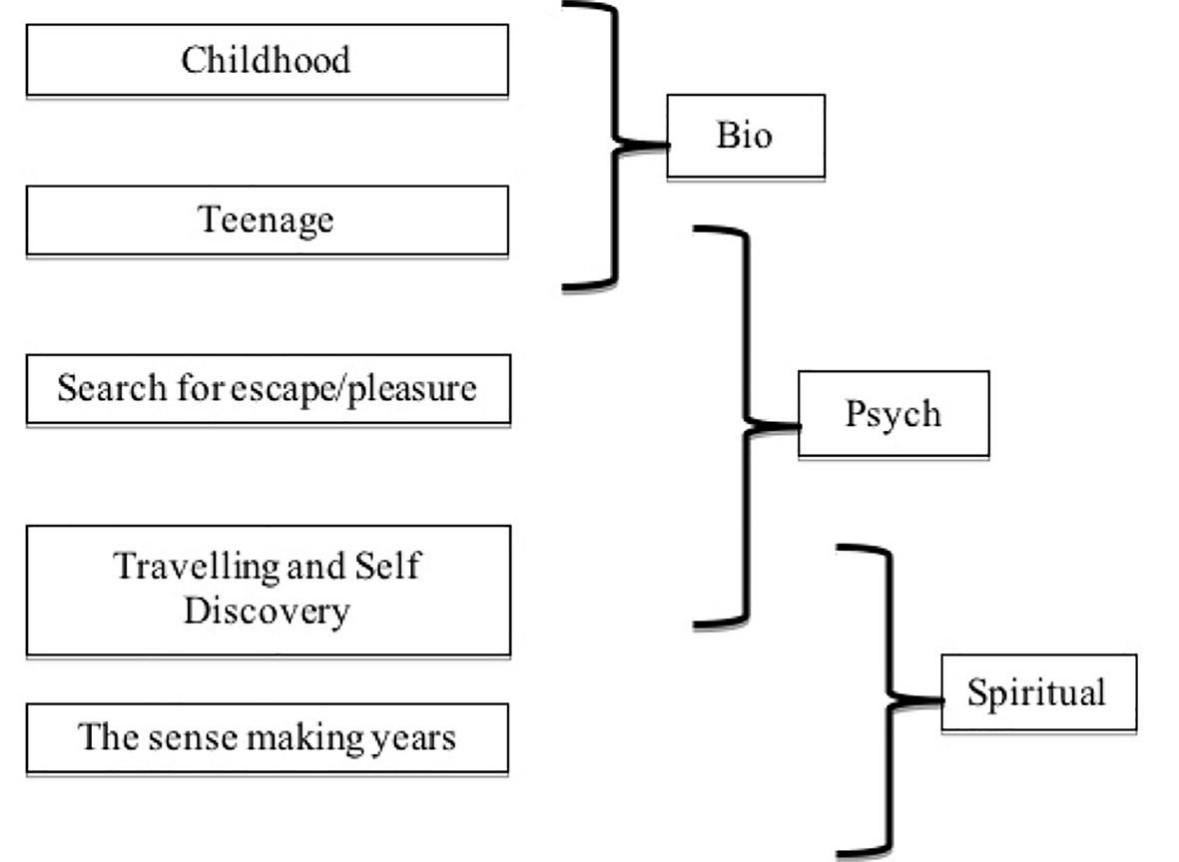
Maya Angelou’s Progression Through the Three Dimensions

Frankl proposed that individuals move through the different dimensions throughout their lives, and this appears to have been true of Angelou’s progression in the first 40 years of her life. Beginning in childhood, she moved from a focus on the biological dimension, through the psychological dimension before finally embracing the spiritual dimension in her early 40s as she moved into travelling and self-discovery. This progression is presented in Figure 1 below and discussed in more detail in the following paragraphs.

Throughout her early life and teenage years, Angelou appears to have been absorbed with her body and her physicality. This is apparent in the opening sections of her first autobiography, where she writes of an intense dissonance between her sense of self and her body: “Wouldn’t they be surprised when one day I woke out of my black ugly dream … [I was] … a too-big Negro girl, with nappy black hair, broad feet and a space between her teeth” (Angelou, 2004, p. 8). In combination with her dislike of her physical appearance, Angelou also experienced an intense need for physical affection and intimacy that was unmet during her childhood. Angelou was never hugged or held by her grandmother (referred to as Momma) who was embarrassed by any show of emotion that was not directly linked to religion. Angelou recalled that her grandmother would have “been more surprised than I had she taken me in her arms … Her world was bordered on all sides with work, duty, religion” (Angelou, 2004, p. 47). The lack of physical affection became linked to her dislike of her body, which made her almost desperate for validation and connection. “I wasn’t pretty or even cute. That woman who looked like a movie star deserved a better looking daughter than me.” (Angelou, 2013, p. 14). During this stage in her life, the young Angelou equated good looks with worthiness and believed that her physical appearance made her unworthy of receiving love.

The entrenchment within the biological and psychological dimensions continued throughout childhood and was compounded by Angelou’s experience of childhood sexual abuse. Angelou was traumatised by the abuse and was confused by the fact that she also enjoyed the physical intimacy and longed for it (Angelou, 2004), as it was lacking in her other relationships. This event defined Angelou’s relationships with men through most of her adolescent and early adult years and led to Angelou developing distorted views on love and sex. In these relationships, young Angelou would completely ignore her own feelings in favour of those of her partner: “back in the car, it never occurred to me to put up resistance…I wanted to do what he wanted, so I sat quiet” (Angelou, 2004, p. 241). Angelou wanted to completely immerse her entire being into her relationships and had a poor sense of self as she mentioned that “The naturally lonely person does not look for comfort in love, but accepts the variables in due course” (Angelou, 2004, p. 341). The most extreme example of this occurred when Angelou willingly worked as a prostitute at the request of her partner.

As she moved into adolescence and early adulthood, Angelou continued to experience unhappiness with her physical appearance, but this became increasingly linked to psychological conflicts related to trying to clarify her identity and roles. She became fixated on certain aspects of herself, such as her need to be a perfect mother and her striving for a dance career. She experienced extreme guilt over her abandonment of Guy (her only child) but was simultaneously intent on ignoring this in her search for temporary happiness. This resulted in her becoming depressed and contemplating suicide (Angelou, 2004). “… I can’t see any reason for living … I’m so unhappy. And I have done such harm to [Guy]” (Angelou, 2004, p. 611). Angelou’s psychological conflicts (see Frankl, 1965; Wessels, 2013) were manifested in her continued determination to prove herself to others and her desire to be seen as perfect, admired, and successful. She desperately wanted love, belonging, and acceptance (Angelou, 2004) and believed that she needed to act a certain way in order to achieve these things. Her need for acceptance resulted in her acting in ways that were inauthentic: “I had spent so many years being people other than myself …” (Angelou, 2004, p. 240).

Angelou’s movement away from the biological and psychological dimensions and into the spiritual dimension appears to be marked by two themes. Firstly, her discovery of herself as a dancer allowed her to reconcile with the physical dimension and secondly, her discovery of her voice through writing appears to have allowed her to enter the spiritual dimension in her ability to find meaning through self-expression. Towards the end of the period covered in her autobiographies, as she approached her 40s, Angelou began to be more accepting towards herself as she wrote that “Love was what I had been waiting for. I had done grown up things out of childish ignorance or juvenile bravado, but now I began to mature” (Angelou, 2004, p .242). Angelou’s description of love (Angelou, 2004, 2013) appears to mark her transcendence beyond self-absorption into the spiritual dimension (see Frankl, 1969; Morgan, 2012) and she began to start working through her conflicts. She stopped focusing on her image and fearing rejection and moved to embracing the love of family and friends as well as finding ways, through racial activism and her career, to share this love. Based on her ability to reflect back on her early years in her autobiographies, it would appear that this ability to make meaning within the spiritual dimension persisted into the remainder of her life and career.

Reflecting back on her earlier life in one of her autobiographies, Angelou (2013) wrote:

… the ship of my life might or might not be sailing on calm seas. The challenging days of my existence might or might not be bright and promising … I maintain an attitude of gratitude. If pessimism insists on occupying my thoughts, I remember there is always tomorrow. Today, I am blessed (p. 137).

This quote epitomises the journey described in this analysis, where Angelou moved away from the physical and psychological crises that typified her early life and young adulthood and towards a more spiritual dimension.

Her youthful search for hedonism gave way to a search for purpose and fulfilment in her life. She began to feel uncomfortable about her life and these tensions signalled her awareness of meaning. “I decided that the time had come to stop my dangerous habits like smoking, drinking and cursing … Imagine I might really become somebody. Someday.” (Angelou, 2013, p. 81). Angelou wanted to transcend beyond herself by helping, loving, and educating others. Once she found her purpose in her career and love and security within her family, Angelou felt comfortable within herself. She had acknowledged that meaning existed, she was motivated to discover meaning, and she believed that she was free to discover it no matter her circumstances (Frankl, 1985). It is interesting that Angelou chose to conclude her autobiographical recount at this point, as it clearly indicated a point at which she felt that a phase of her life had been completed.

### Fundamental Triad

Unlike the tri-dimensional view of humans where Angelou clearly progresses through the various dimensions of being, there is no clear chronological development of meaning in Angelou’s life as recounted in her autobiographies. Instead, she appears to have engaged with each of the three components of the triad throughout the period of her adult life (age approximately 20 to 40) recounted in the autobiographies, developing an increasing awareness of and engagement with each aspect of meaning as her life progressed. This may be because the account we have of her meaning making is retrospective (in the form of autobiographies) and so the very process of writing for Angelou constituted a discovery of meaning. However, although there is no chronological development it is still possible to trace each aspect of the fundamental triad within Angelou’s life narrative.

Angelou’s awareness of the existence of meaning was a gradual process. She was aware of the existence of God from childhood but did not have a spiritual connection until her early to mid-adulthood years. Although young Angelou grew up in a religious home and attended church frequently, she was cynical about religion. During her teenage years, Angelou was disconnected from religion and focussed on her career and academics. Angelou initially became aware of meaning not through organised religion, but through the realisation that there was a purpose and a fulfilment to be sought through a career. Angelou’s reaction to her rejection by the Army “… My life has no centre, no purpose…” (Angelou, 2004, p. 308) was an early foreshadowing of the meaning she attached to her career. She went on to discover meaning throughout her involvement in her careers as a dancer, activist and writer – she wrote that “dancing liberated me and even made me feel as if my body had a reason to be” (Angelou, 2013, p. 110). Her involvement in racial politics connected her to the discovery of meaning as she was able to transcend beyond herself to be concerned about other people. Angelou was aware of the tensions within herself and this caused her to search for something beyond herself as is evident in the various movements in her career throughout her life: “My position had always been that no one was responsible for my life except me.” (Angelou, 2004, p. 755). Although the autobiographies do not cover the later years of Angelou’s life, it seems likely that her career as a writer allowed her to discover meaning through her writing as she reflected on her life. Thus, in adulthood she appears to have experienced a settling down and an increased spirituality and connection with others (Agins, 2013; Angelou, 2004; Frankl, 1969).

The second component of the fundamental triad, the will to meaning, is evident throughout Angelou’s narrative. As a child, she refused to believe in the passivity of Momma and the people in Arkansas. Instead, she believed that the racial system could be changed, and she was determined to do something about it. One of the first instances of her asserting herself was with her employer Mrs Cullinan: “For a week I looked into Mrs Cullinan’s face as she called me Mary … When I heard Mrs Cullinan scream, “Mary!” I picked up the casserole and … the green cups … I let them fall on the tiled floor” (Angelou, 2004, p. 87). This became constant in her life and was especially prominent during the years when she was involved in Black activism (Agins, 2013; Angelou, 2004). It is important to note the shift in Angelou’s will to meaning. Her assertations and defiance against the entrenched racial political system were initially reactive and involved working through psychological conflicts related to her insecurities surrounding race. Her will to meaning arose from her understanding of the system where “The historically oppressed can find not only sanctity but safety in the state of victimisation. When access to a better life has been denied often enough … one can use the rejection as an excuse to cease all efforts.” (Angelou, 2004, p. 461). For Angelou, this thinking indicated a shift in how she perceived race and relations, where she started to increasingly assert that black people should not just accept a victim identity but instead should fight for their place in society.

Another theme that provides evidence of Angelou’s will to meaning was her involvement in her career. She wanted a career that would fulfil her and give her purpose. She tried several careers throughout her lifetime, each time seeking a career that would provide her with purpose and make her feel fulfilled. She enjoyed dancing and writing and this allowed her to transcend biological and psychological issues and fully engage with life and the meaning making process (Angelou, 2004). She knew that she was free to make her own choices and was also responsible for them (Frankl, 1967, 1969; Meyer, 1997).

The third component of the fundamental triad is freedom to will, where individuals are free to explore their lives in the discovery for meaning but must also accept responsibility for every choice that they make (Frankl, 1985). Angelou believed that it was important to explore and experience life to the fullest. She wanted to be active and engaged in all aspects of her life and was especially vehement against the racial system. She believed that she was free to act against the racial systems imposed on Black people (Lupton, 1998). She was involved in organisations during adulthood as chronicled in her autobiographies and felt free to question her entrenched racial assumptions. Her freedom to will is evident in her movement towards settling down and accepting herself as she realised that “As always, again. We survived. The depths had been icy and dark, but now a bright sun spoke to our souls … I was a proud member of the wonderful, beautiful Negro race.” (Angelou, 2004, p. 143). She began to embrace meaning making in her life as she discovered the freedom within her to do so, as well as accepting the responsibility for her own life. Angelou took responsibility for her choices and felt that she had to carry out racial activism in a responsible manner (Agins, 2013; Angelou, 2004, 2013; Frankl, 1985). Angelou also felt responsible for her career; she believed that she had the freedom to choose the jobs that she wanted and the responsibility to be the best she could in these jobs. Racial politics and her career were always a freedom to will for Angelou and motivated her throughout her narrative. Angelou believed that, in general, she was responsible for any choices or decisions that she made, even those that were considered mistakes. This included her becoming pregnant with Guy:

For eons, it seemed, I had accepted my plight as the hapless, put-upon victim of fate and the Furies, but this time I had to face the fact that I had brought my new catastrophe upon myself … so I hefted the burden of pregnancy at sixteen onto my own shoulders where it belonged (Angelou, 2004, p. 218).

Her responsibility moved to responsibleness where she felt free to carry out her purpose and therefore have freedom in meaning making. A poignant quote by Angelou perfectly summarises her engagement with life and the importance of meaning making:

… how did I get to be Maya Angelou … I knew that I had become the woman I am because of my grandmother I loved and the mother I came to adore … Love heals … a condition so strong that it may be that which holds the stars in their heavenly positions … This book has been written to examine some of the ways love heals and helps a person to climb impossible heights and rise from immeasurable depths (Angelou, 2013, p. x).

### Limitations and Direction for Future Research

The most notable limitation of this study is the specific focus on meaning making, which precluded the discussion of other aspects of Angelou’s life (e.g. her literary achievements, her activism). Future research should aim to utilise multiple theoretical lenses, such as those that explore creativity or social change, to offer a more comprehensive portrait of this extraordinary life. This study did indicate that Frankl’s theory provides a useful lens through which to analyse the development of meaning in an individual life and as such it is suggested that future research in psychobiography make use of this theory.

A further limitation is the focus on the first 40 years of Angelou’s life. This limitation is based on the period of time covered in Angelou’s autobiographies and as such is inevitable due to the nature of the available data. Future research may wish to expand the data set by including more extensive biographies and perhaps also interviews with Angelou’s friends and family to develop a portrayal of meaning making in the period of Angelou’s life not covered by her autobiographies.

## Conclusion

The primary aim of the study was to explore and reach an understanding of meaning making in the early life and young adulthood of Maya Angelou, as illustrated in her autobiographies, through the application of Frankl’s existential psychology. The article utilised two major concepts within Frankl’s theory: that of the tri-dimensional nature of humans and the fundamental triad. The tri-dimensional nature of humans indicated Angelou’s movement from the biological dimension, through the psychological and achieving meaning making in the spiritual dimension. Her childhood, adolescent and early adult years were defined by biological concerns and psychological conflicts. She was able to come to terms with these and move towards transcendence into a spiritual dimension of existence (Frankl, 1967, 1969), which she seems to have reached by the time that her autobiographical narrative ceases. The fundamental triad draws from and builds on this view of humanity, as there needs to be an internal striving toward meaning making before understanding how meaning making works in a human life. Maya Angelou worked through insecurities and had become aware of meaning during her adult years and had an active will to meaning and freedom to will. She was motivated to create and to educate. She wanted to tell people about her experiences and struggles so that they could learn from them (Lupton, 1998). She wanted to spread love and peace across the world. She had the freedom to choose her attitude and accepted responsibility for her life. She chose to do something worthy and purposeful with her life (Agins, 2013; Angelou, 2013; Frankl, 1985).
